# Increased Testosterone Decreases Medial Cortical Volume and Neurogenesis in Territorial Side-Blotched Lizards (*Uta stansburiana*)

**DOI:** 10.3389/fnins.2017.00097

**Published:** 2017-03-01

**Authors:** Lara D. LaDage, Timothy C. Roth, Cynthia J. Downs, Barry Sinervo, Vladimir V. Pravosudov

**Affiliations:** ^1^Division of Mathematics and Natural Sciences, Penn State University AltoonaAltoona, PA, USA; ^2^Department of Psychology, Franklin and Marshall CollegeLancaster, PA, USA; ^3^Biology Department, Hamilton CollegeClinton, NY, USA; ^4^Department of Ecology and Evolutionary Biology, University of CaliforniaSanta Cruz, CA, USA; ^5^Department of Biology, University of NevadaReno, NV, USA

**Keywords:** testosterone, neurogenesis, medial cortex, dorsal cortex, lizards, *Uta stansburiana*

## Abstract

Variation in an animal's spatial environment can induce variation in the hippocampus, an area of the brain involved in spatial cognitive processing. Specifically, increased spatial area use is correlated with increased hippocampal attributes, such as volume and neurogenesis. In the side-blotched lizard (*Uta stansburiana*), males demonstrate alternative reproductive tactics and are either territorial—defending large, clearly defined spatial boundaries—or non-territorial—traversing home ranges that are smaller than the territorial males' territories. Our previous work demonstrated cortical volume (reptilian hippocampal homolog) correlates with these spatial niches. We found that territorial holders have larger medial cortices than non-territory holders, yet these differences in the neural architecture demonstrated some degree of plasticity as well. Although we have demonstrated a link among territoriality, spatial use, and brain plasticity, the mechanisms that underlie this relationship are unclear. Previous studies found that higher testosterone levels can induce increased use of the spatial area and can cause an upregulation in hippocampal attributes. Thus, testosterone may be the mechanistic link between spatial area use and the brain. What remains unclear, however, is if testosterone can affect the cortices independent of spatial experiences and whether testosterone differentially interacts with territorial status to produce the resultant cortical phenotype. In this study, we compared neurogenesis as measured by the total number of doublecortin-positive cells and cortical volume between territorial and non-territorial males supplemented with testosterone. We found no significant differences in the number of doublecortin-positive cells or cortical volume among control territorial, control non-territorial, and testosterone-supplemented non-territorial males, while testosterone-supplemented territorial males had smaller medial cortices containing fewer doublecortin-positive cells. These results demonstrate that testosterone can modulate medial cortical attributes outside of differential spatial processing experiences but that territorial males appear to be more sensitive to alterations in testosterone levels compared with non-territorial males.

## Introduction

Animals that defend clearly delineated territories are in part reliant upon spatial memory to remember territorial boundaries and determine the location of territorial neighbors (Falls, 1982; Godard, 1991; McGregor and Westby, 1992; Sherry, 1998; Bee and Gerhardt, 2001). However, territory size can vary among individuals suggesting that individuals likely have differential demands on spatial cognitive processing based on the size of the defended territory. In support of this, previous studies in a variety of taxa have demonstrated that individuals that hold territories, hold larger territories, or traverse larger home ranges have better spatial memory. Further, these individuals also have larger hippocampi, the area of the brain largely responsible for spatial memory processing, with more new hippocampal neurons, presumably to subserve the increased demands on spatial cognition (Gaulin and Fitzgerald, 1986, 1989; Jacobs et al., 1990; Galea and McEwen, 1999; Amrein et al., 2004; Roth et al., 2006; LaDage et al., 2009, 2013; Holding et al., 2012). Consequently, there is a positive relationship among territoriality, spatial area use, spatial cognition, and the underlying neural substrate supporting spatial cognition.

What remains unclear is the mechanism underlying the correlation among territoriality, spatial cognition, and the brain. During the breeding season, territorial behavior and territory size have been linked to testosterone, in that increased levels of testosterone correlate with increased territory size and improve spatial cognition in mammals, birds, and lizards (e.g., Wingfield, 1984; DeNardo and Sinervo, 1994; Moss et al., 1994; Sinervo et al., 2000; Veiga et al., 2001; Ketterson et al., 1996; Spritzer et al., 2011). Variation in hormone levels can also directly affect the hippocampus and these changes can be maintained long-term in mammals (e.g., Gould et al., 1990; Roof and Havens, 1992; Woolley and McEwen, 1992; Cooke et al., 1998; Galea et al., 1999). Specifically, in birds and mammals, testosterone levels correlate with increased brain substructure volume and increased neurogenesis rates (e.g., Roof and Havens, 1992; Absil et al., 2003; Galea et al., 2006). While testosterone has been linked to both territoriality and hippocampal attributes, it remains unclear if testosterone is the underlying mechanism that links the two and, if so, whether variation in testosterone can directly alter brain attributes, outside of spatial area use experiences.

While many of the aforementioned studies have leveraged naturally occurring hormonal changes across seasons, between the sexes, or across species to examine these relationships, there are genetic, population-level, and other life history variables that can introduce variance not attributable to differences in neuroendocrinology (e.g., Knapp, 2003; Moore, 1991; LaDage, 2016). One way to circumvent some of these issues is to utilize model species that demonstrate polymorphisms within a sex. Polymorphisms allow individuals to engage in different strategies to increase reproductive success and also correlate with differences in other dimensions, such as morphology, physiology, and other life history characteristics (e.g., Sinervo et al., 2000; Crews and Moore, 2005). Thus, the use of a polymorphic species allows for naturally occurring intrasexual differences yet controls for potentially confounding variables associated with intersexual, population-level, or interspecific differences not related to the neuroendocrinology axis. This approach has been relatively successful, particularly in reptiles, and much research has been directed toward understanding the hormonal influences on the organization and activation of physiological, morphological, and behavioral differences within polymorphic species (e.g., Moore, 1991).

In the current study, we used side-blotched lizards (*Uta stansburiana*), a polymorphic species in which male morphotypes differ in space use, hormonal profile, and brain attributes. In our target population, males are found in one of the three morphotypes and all morphs are genetically determined by a single locus (OBY locus) (orange morph: *oo, bo, yo*; blue morph: *bb*; yellow morph: *by, yy*) (Sinervo et al., 2001, 2006). Each morph has a different space use strategy: the orange morph occupies and defends large territories, the blue morph occupies and defends smaller territories while the yellow morph does not hold or defend a territory, and its home range is smaller than the territories of the other two morphs (Sinervo and Lively, 1996; Sinervo et al., 2000, 2006; Zamudio and Sinervo, 2000).

Territorial predisposition in side-blotched lizards is associated with differences in territory size, testosterone profiles, and cortical attributes. Previous studies in this and other polymorphic lizard species have found that territorial morphotypes have higher levels of testosterone than non-territorial males (e.g., DeNardo and Sinervo, 1994; Wikelski et al., 2005; Olsson et al., 2007) and this is one mechanism that can directly modulate spatial area use. For example, experimentally elevating testosterone levels in non-territorial males induces an increase in home range size similar to home range sizes seen in un-manipulated territorial males (DeNardo and Sinervo, 1994; Sinervo et al., 2000; Wikelski et al., 2005) suggesting that testosterone is sufficient to regulate spatial area use.

However, whether testosterone is the modulating mechanism underlying changes in spatial area use and the brain is still unknown. Our previous research indicates that the putative reptilian hippocampal homologs, the dorsal and medial cortices (Butler, 1976), are larger in territorial than non-territorial males (LaDage et al., 2009) yet these differences are not fixed and can vary based on environmental experiences (LaDage et al., 2013, 2016). Further, territorial predisposition can modulate the effects of environmental experiences on cortical plasticity in that only territorial males upregulate neurogenesis in the face of increased spatial area use, possibly due to territorial males' higher reliance on spatial processing (LaDage et al., 2013). Thus, known factors that influence neural plasticity can also differentially modulate that plasticity based on territorial status.

Since there is a relationship between testosterone and the brain, as well as between testosterone and territoriality, and if testosterone is the mechanistic mediator between territorial predisposition and the brain, experimentally elevating testosterone levels should increase cortical attributes, independent of spatial use experiences. We predicted that, in individuals hatched and raised with identical spatial area experiences, testosterone supplementation would increase cortical attributes in all males, regardless of territorial predisposition. Further, because territorial predisposition has been found to modulate cortical attributes, we also predicted that territorial males would be more sensitive to changes in testosterone because territorial males are more dependent on spatial cognition within the context of territorial defense. Thus, while testosterone may upregulate cortical attributes in both territorial and non-territorial males, the effects should be more dramatic in territorial males.

## Materials and methods

### Subjects

In March 2010, adult male and non-gravid female side-blotched lizards were collected near Los Baños Grandes, California and transported to the University of Nevada, Reno. Morphotypes were determined by external morphological characteristics (e.g., Sinervo and Lively, 1996) in that homozygote males have solid throat colors: (*oo*) males have purely orange throats, blue males (*bb*) have purely blue throat coloration, and yellow males (*yy*) have pure yellow throats. Because male throat coloration easily identifies morphotype, males and females were assigned to breeding enclosures (20-L terraria) to facilitate the production of primarily homozygous morphotypic offspring (LaDage et al., 2013, 2016). Females were subjected to abdominal palpitations every other day and gravid females were transferred to individual ovipositing enclosures until laying [enclosure size: 22 × 14 × 13.5 cm with a moist layer of peat/sand (3:1)]. Ovipositing enclosures were checked for eggs every morning and, after oviposition, females were returned to their breeding enclosure.

Eggs were collected from ovipositing enclosures and individually incubated at 28 ± 1°C until the emergence of hatchlings (35–45 days, L. LaDage, personal observation). To prevent pseudoreplication, only one male offspring per mated pair was used in the current experiment. Male hatchlings were individually housed (enclosure size: 22 × 14 × 13.5 cm), fed live crickets dusted with calcium and vitamins each day, and supplied with water *ad libitum*. The temperature of the laboratory was maintained at 20°C and cages were supplemented with above-cage basking lights which provided a thermal gradient from 25 to 40°C. All lights were kept on a cycle mirroring ambient across the year (e.g., 10L:14D in December, 16L:8D in June). Individuals were raised until adulthood (>9 mo) before being subjected to the experimental treatment thus assessing the activational role of testosterone rather than the organizational (Moore, 1991). All procedures were approved by the Institutional Animal Care and Use Committee at the University of Nevada, Reno (2009-00434) in accordance with the guidelines of the American Veterinary Medical Association.

### Experimental protocol

Our previous studies indicate no neurobiological differences in cortical volume between orange and blue territorial males (LaDage et al., 2009, 2013) suggesting that brain attributes are likely regulated by how morphotypes use space rather than the size of the space *per se* (e.g., Clint, 2012). Thus, we collapsed morphotypes into territorial predisposition, with orange and blue males comprising the territorial group and yellow males comprising the non-territorial group. We randomly assigned territorial and non-territorial males to either a control or testosterone-supplemented treatment group (*n* = 12 for territorial, *n* = 9 for non-territorial at the conclusion of the study). A 3 mm length of Silastic medical grade tubing (Dow Corning 602–305) was filled with saline (control group) or 1 mm testosterone (Sigma T1500) (supplemented group). Implants were sealed at each end with silicone sealant and soaked in saline for 24 h before implantation (DeNardo and Licht, 1993; DeNardo and Sinervo, 1994). All subjects were subcutaneously anesthetized with 0.2% lidocaine, a small incision was made in the flank, and the implant was placed intracoelomically through the incision (DeNardo and Sinervo, 1994). These implants have been shown to maintain elevated levels of testosterone for at least 3 months in this species (DeNardo and Licht, 1993; DeNardo and Sinervo, 1994). After implantation, subjects were returned to their home enclosures for 2 months, which allowed for an adequate amount of time in which testosterone could possibly affect cortical attributes (e.g., Delgado-Gonzalez et al., 2011). Further, because implantation occurred in April, all males were well into breeding age and, in their natural habitat, would have exhibited increased levels of testosterone and territory establishment/defense (e.g., Ferguson and Fox, 1984; Wilson and Wingfield, 1994).

### Testosterone assays

To assess baseline and terminal testosterone levels, as well as the success of our implants in elevating testosterone, we collected blood in all subjects the week before implantation and at the time of sacrifice. Blood was collected via the retro-orbital sinus using two to three 20 ul heparinized microcapillary tubes and kept on ice for no more than 3 h. We centrifuged samples to isolate the plasma and froze plasma samples at −80°C until processed (<3 months). We quantified testosterone concentrations (pg/ml) using a commercially available ELISA kit (#ADI-900-065, Enzo, Farmingdale, NY 11735) (Robertson et al., 2011). Prior to use, we validated the kits for *Uta* by created a dilution curve of unknown samples (Buchanan and Goldsmith, 2004); we found parallelism between standards and *Uta* samples. All samples were quantified using kits from the same lot, and we used the protocol included with the kit. End absorbances were quantified on a 405 nm plate reader (Thermo Scientific Multiskan Ascent). Samples were tested in duplicate at the predetermined optimal dilution of 1:20. Because some samples had testosterone levels that were outside the standard curve, we reassayed these samples at more dilute concentrations until they fell within the ideal part of the standard curve; repeated assays were at either 1:40 or 1:500.

### Tissue processing

After 2 months in their respective treatment groups, all individuals were anesthetized with a lethal overdose of Nembutal (510 mg/kg of 50 mg/ml sodium pentobarbital), then transcardially perfused with 0.1 M phosphate buffered saline for 10 min followed by a 15–20 min perfusion of 10% methanol-free formalin (from paraformaldehyde). Brains were extracted and post-fixed for 24 h in 10% methanol-free formalin (from paraformaldehyde), cryoprotected in 15% sucrose for 24 h, 30% sucrose for another 24 h, and finally flash-frozen on dry ice. Brains were stored at −80°C until sectioning. Brains were sectioned in the coronal plane every 40 μm (Leica CM 3050S, −20°C). Sections were divided into two series. One series was mounted and Nissl-stained with thionin to visualize cortical boundaries while the second series was subjected to immunohistochemistry to visualize the production of new neurons (e.g., LaDage et al., 2013, 2016).

### Immunohistochemistry

To visualize new neurons, the second series of tissue sections were processed for the expression of doublecortin, an endogenous protein expressed by immature, migrating neurons, which co-labels with other markers specific to new neurons (Brown et al., 2003; Rao and Shetty, 2004; Couillard-Despres et al., 2005; Hairston et al., 2005; Balthazart et al., 2008; Luzzati et al., 2009; Delgado-Gonzalez et al., 2011). In lizards, doublecortin expression in new neurons lasts between 7 and 90 days, although 2 weeks to 30 days is more typical (Lopez-Garcia et al., 1990; Ramirez-Castillejo et al., 2002; Marchioro et al., 2005; Delgado-Gonzalez et al., 2011). After these neurons migrate and become incorporated into the existing neural architecture, expression of doublecortin ceases while expression of mature neuronal proteins begins (Mullen et al., 1992; Brown et al., 2003). Thus, in lizards, quantifying doublecortin protein expression encompasses the majority of the neurons in the immature stages of development.

To visualized doublecortin expression, sections were washed in tris (hydroxymethyl) aminomethane-buffered saline (TBS), incubated in 30% hydrogen peroxide plus TBS (1:50) at room temperature for 30 min, washed in TBS, incubated in blocking buffer (normal horse serum, 1:33.3; Triton X-100, 1:39.8; and TBS) at room temperature for 30 min, and then incubated in anti-doublecortin antibody plus blocking buffer (1:200; Santa Cruz Biotechnology, Santa Cruz, CA; SC-8066) overnight (approximately 18 h) at 4°C. The following day, sections were washed in TBS, incubated in biotinylated horse anti-goat antibody in blocking buffer (1:200; Vector Laboratories, Burlingame, CA; BA-9500) at room temperature for 2 h, washed in TBS, incubated in a Vectastain Elite ABC kit (Vector Laboratories, PK-6100) at room temperature for 1 h, washed in TBS, reacted with diaminobenzidine-nickel kit (DAB-Ni; Vector Laboratories, SK-4100) at room temperature for 1 min and again washed in TBS and mounted on slides. We also performed a negative control to account for nonspecific binding of the secondary antibody. To do so, we repeated this protocol, but replaced the anti-doublecortin antibody with TBS during overnight incubation. The elimination of the antibody suppressed staining, thus indicating that our protocol was specifically staining cells expressing doublecortin.

### Brain analysis

In the reptilian brain, the medial and dorsal cortices exhibit structural and functional homologies to the hippocampus of other vertebrates (e.g., Grisham and Powers, 1990; Reiner, 1991, 1993; Butler, 1994; Petrillo et al., 1994; Reiman Avigan and Schade Powers, 1995; Striedter, 1997; Rodríguez et al., 2002; López et al., 2003; Striedter, 2016) thus both areas were measured in their entirety. We used standard stereological methods (StereoInvestigator, Microbrightfield, Inc., Williston, VT; Leica M4000B microscope) to estimate the remainder of the telencephalon (total telencephalon minus the dorsal and medial cortical volumes), the dorsal cortex, and the medial cortex volumes in nissl- stained slides, as well as production of new neurons in the dorsal and medial cortices in doublecortin-stained slides. All methods have been previously optimized for this species (LaDage et al., 2009, 2013, 2016) and all coefficients of error were < 0.10 (Gundersen et al., 1999).

**Figure 1:**
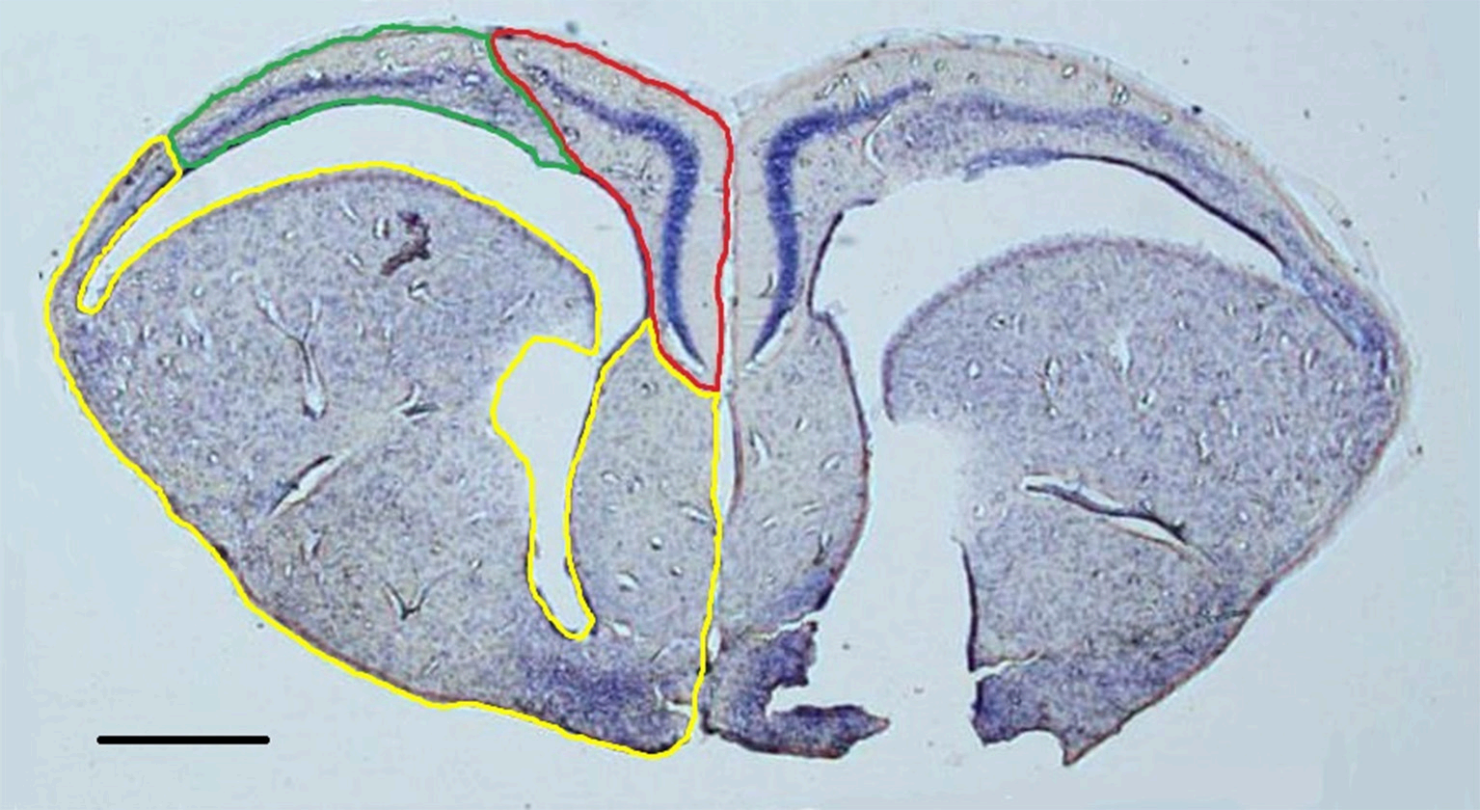
**Representative nissl-stained section (1.25x) with the medial cortex (red), dorsal cortex (green), and remainder of the telencephalon (yellow) outlined in the left hemisphere**. Both hemispheres were contoured in order to construct volumetric estimations. Scale bar = 500 μm.

For nissl-stained slides, the left and right hemispheres of the cortical regions and the remainder of the telencephalon were contoured on every section (Figure 1). Volumes were estimated with the Cavalieri procedure (Gundersen and Jensen, 1987) using a 200 μm grid for the cortical regions and a 300 μm grid for the remainder of the telencephalon. Because of the section thickness necessary for stereological estimations of doublecortin-positive cells, the high density of cells within the cell layers of the cortices rendered mature cell bodies indistinguishable (e.g., Ulinski, 1990). Consequently, performing unbiased stereological estimations of total number of neurons was not feasible.

**Figure 2:**
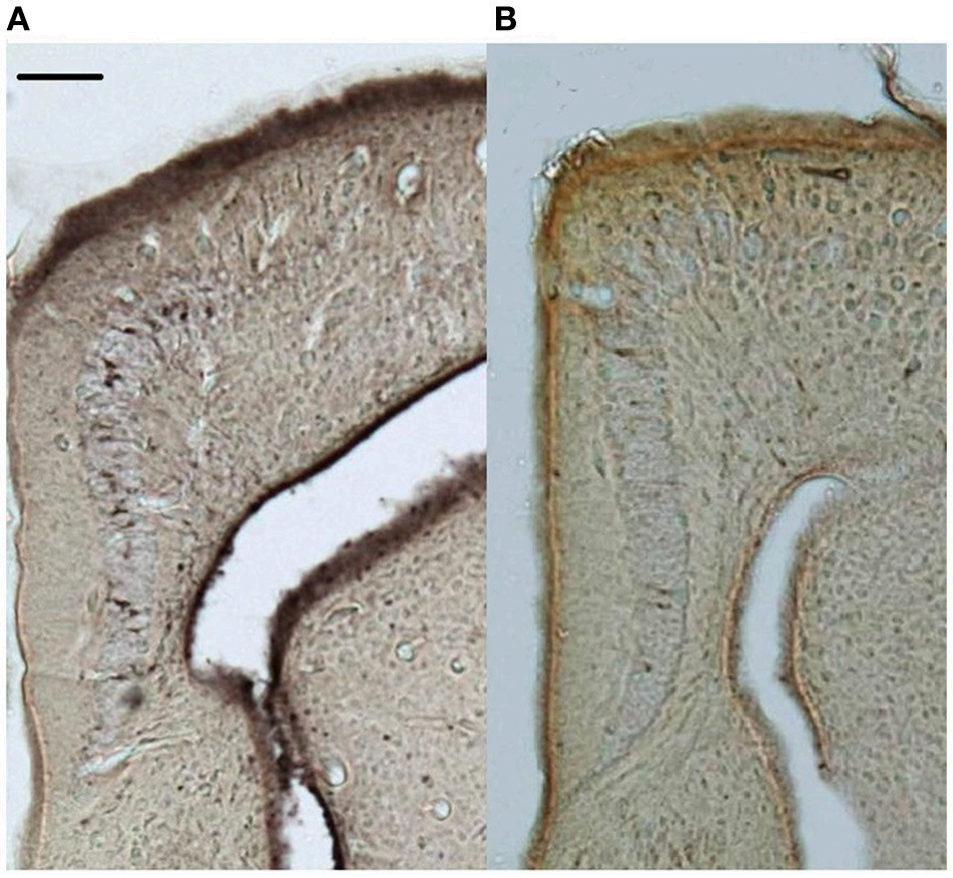
**Representative doublecortin-stained sections (5x) of the medial cortices from (A)** control and **(B)** supplemented territorial male side-blotched lizards (*Uta stansburiana*). Scale bar = 100 μm.

**Figure 3:**
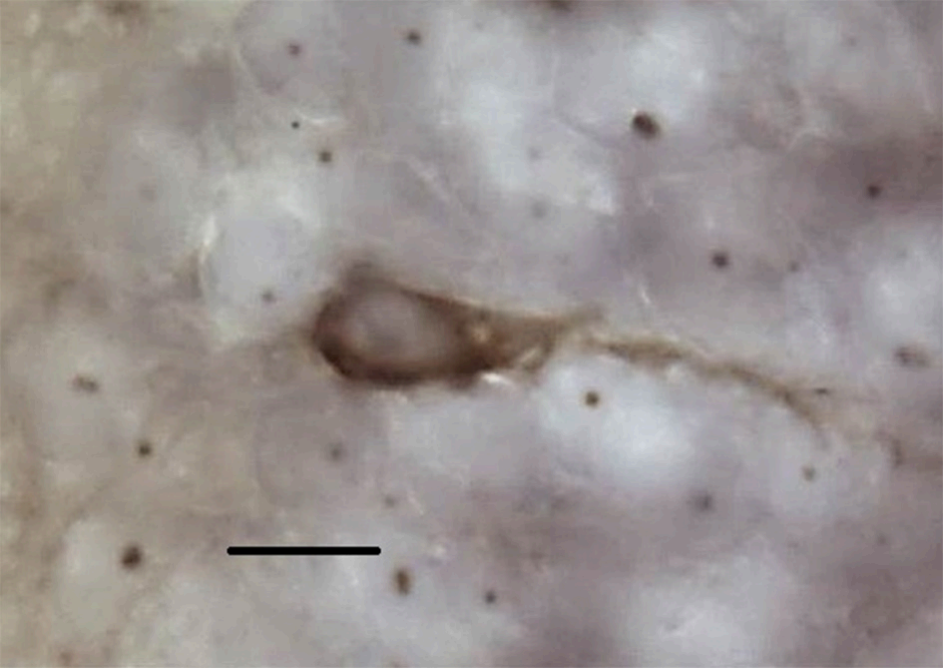
**Representative doublecortin-positive cell at 100x**. Scale bar = 10 μm.

New neuron counts were performed on the doublecortin-stained sections (Figure 2); the left and right hemispheres of the cortical regions were contoured and new neurons were counted exhaustively with the Optical Fractionator procedure (counting frame: 70 × 70 μm, grid: 70 μm, dissector height: 5 μm). All doublecortin-positive cells were counted including migrating fusiform cells typically aligned perpendicular to the ventricular zone as well as the more mature, spherical phenotype (e.g., Pérez-Cañellas and García-Verdugo, 1996; Figure 3). All slides were measured blind to treatment group and territorial identity. Due to histological artifacts, not all brain regions could be analyzed in all animals.

### Statistical methods

There were no differences between the left and right hemispheres for any of the measured variables (paired *t*-tests: all *p* > 0.097) thus data from the left and right hemispheres were summed and subsequent analyses were performed on the pooled data. Homogeneity of variances was assessed with Levene's test and, if the assumption was not met, data were either log or square root transformed to conform to the assumption (*p* > 0.072 for all variables after transformation). Using repeated-measures GLM, we tested for differences in weight and testosterone levels before and after implantation based on territorial predisposition and treatment. We assessed changes in the overall telencephalon volume using GLM with body mass as a covariate to ascertain the effects of territorial predisposition and treatment on overall brain size, outside of changes due to allometry. We also used GLM to assess the effects of territorial predisposition and treatment on the volumes of the dorsal and medial cortices, using remainder of the telencephalon volume as a covariate. Doing so assured that our results were specific to changes in the cortices of the telencephalon, rather than global changes in the brain. Finally, we also used GLM to assess the effects of territorial predisposition and treatment on the production of new neurons in the medial and dorsal cortices, with either dorsal or medial cortex volume as a covariate, essentially assessing new neuron density in these substructures (e.g., LaDage et al., 2013). We also performed analyses without covariates to ascertain any statistical differences when not controlling for the covariates. Testosterone levels did not correlate with changes in brain attributes so was not used as a covariate in the analyses (all *r*^2^ < 0.160, *p* > 0.081). All analyses were conducted with SPSS for Windows, v. 21.0 (IBM Corp.) and we considered results to be statistically significant if *p* < 0.05.

## Results

**Figure 4:**
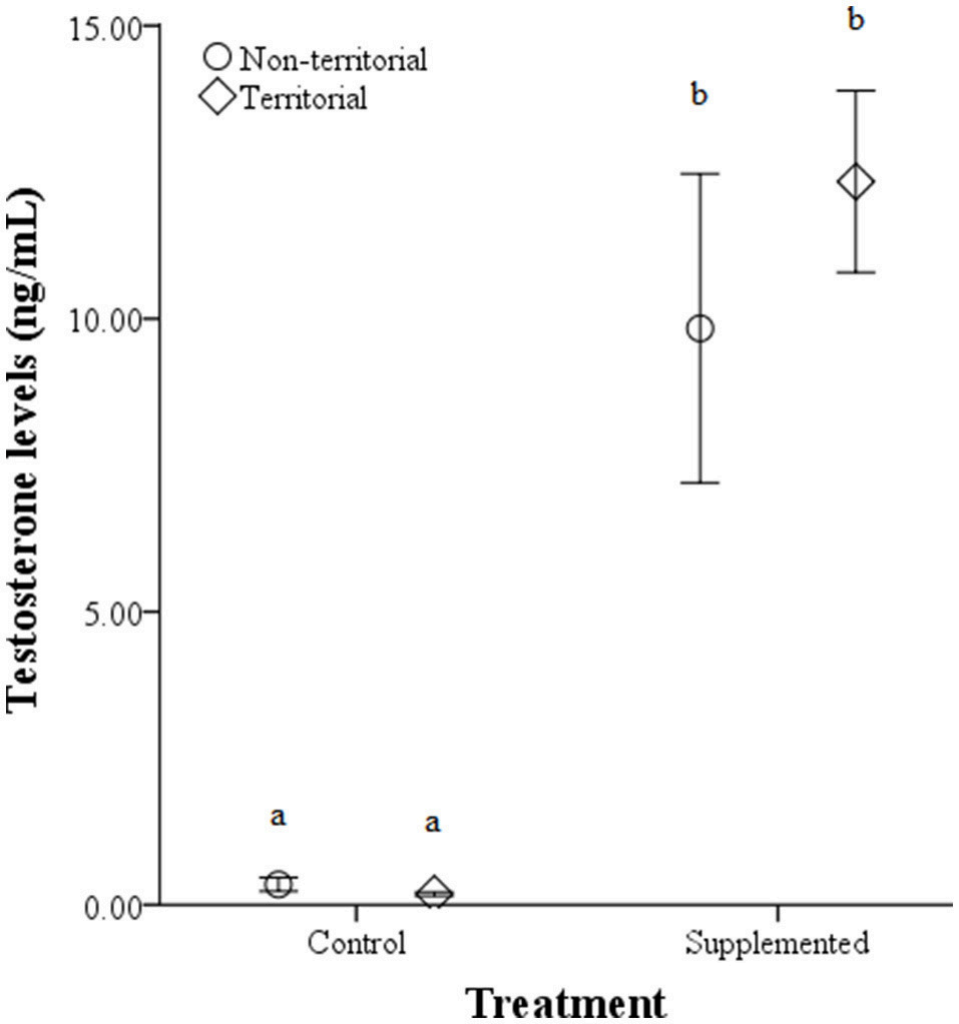
**Circulating testosterone levels (ng/mL) ± SEM in non-territorial (open circles) and territorial (diamonds) male side-blotched lizards (*Uta stansburiana*)**. Individuals were either subjected to testosterone implants (supplemented) or an empty vehicle (control) for 2 months. Implants were sufficient to raise testosterone levels similarly in non-territorial and territorial males. Different letters indicate statistically significant differences (*p* ≤ 0.05).

There were no significant differences in weight before or after implantation between territorial and non-territorial males, control and treatment groups, nor a significant effect of the interaction [territorial predisposition: *F*_(1, 17)_ = 0.292, *p* = 0.596; treatment: *F*_(1, 17)_ = 3.177, *p* = 0.093; interaction: *F*_(1, 17)_ = 0.871, *p* = 0.364]. However, testosterone levels were significantly higher after implantation [*F*_(1, 17)_ = 69.847, *p* < 0.001] and higher in males who received testosterone implants [*F*_(1, 17)_ = 157.321, *p* < 0.001]; territorial predisposition and the interaction among time point, territorial predisposition, and treatment did not significantly affect testosterone levels [territorial predisposition: *F*_(1, 17)_ = 0.106, *p* = 0.748; interaction: *F*_(1, 17)_ = 0.365, *p* = 0.554] (Figure 4).

**Figure 5:**
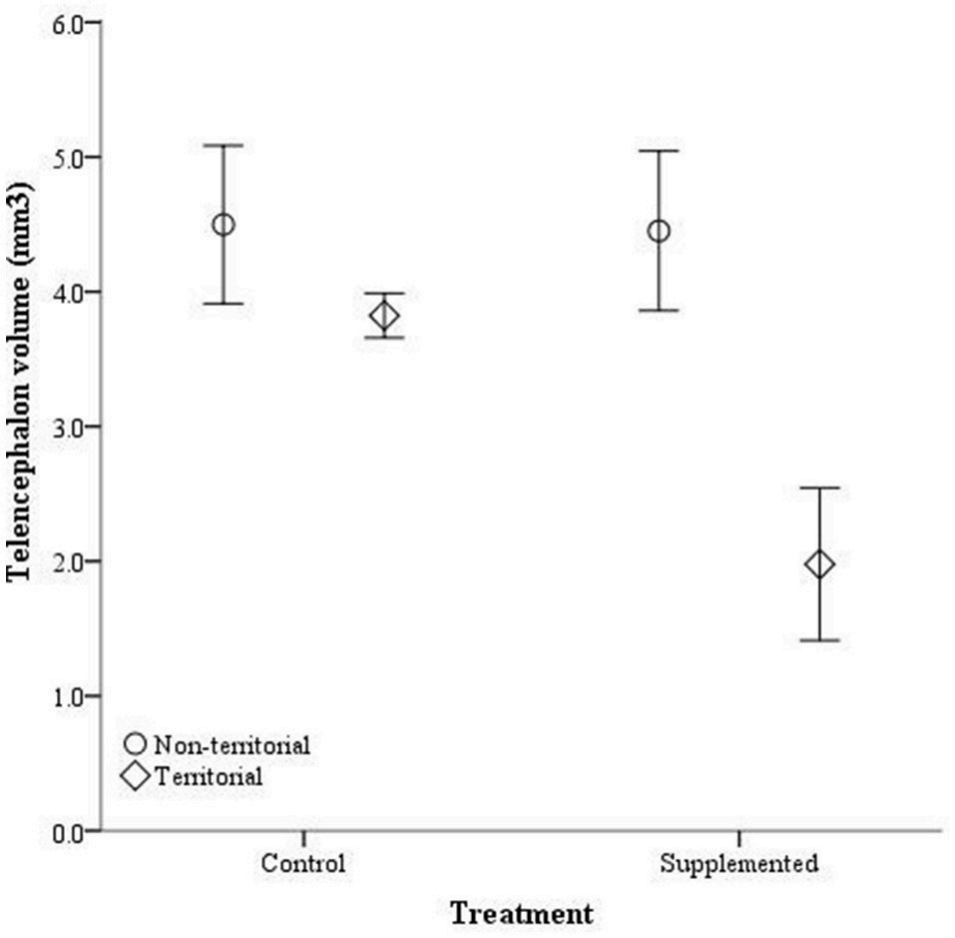
**Absolute telencephalon volume (mm^3^) ± SEM in non-territorial (open circles) and territorial (diamonds) male side-blotched lizards (*Uta stansburiana*)**. Individuals were either subjected to testosterone implants (supplemented) or an empty vehicle (control) for 2 months. Supplemented territorial males had smaller telencephalons than any other group (*p* = 0.013).

**Figure 6:**
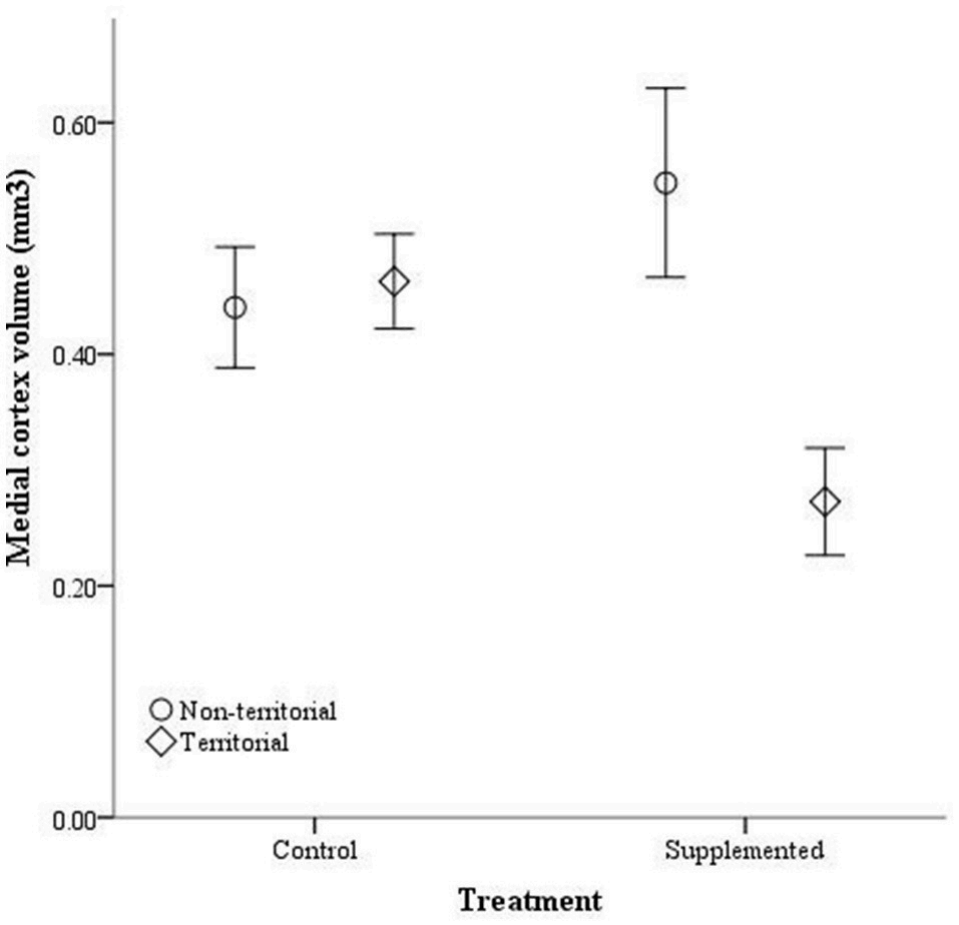
**Absolute medial cortex volume (mm^3^) ± SEM in non-territorial (open circles) and territorial (diamonds) male side-blotched lizards (*Uta stansburiana*)**. Individuals were either subjected to testosterone implants (supplemented) or an empty vehicle (control) for 2 months. Supplemented territorial males had smaller medial cortices than any other group (*p* = 0.014).

After males were subjected to the experimental protocol, telencephalon volume, when controlled for variation in body weight, significantly differed based on the weight covariate [*F*_(1, 14)_ = 6.60, *p* = 0.022], territorial status [*F*_(1, 14)_ = 16.061, *p* = 0.011], and the interaction between territorial status and treatment [*F*_(1, 14)_ = 4.728, *p* = 0.047]; treatment was not significant [*F*_(1, 14)_ = 2.905, *p* = 0.11] (Figure 5). When excluding the body weight covariate in the analysis, we obtained comparable results [territorial status: *F*_(1, 15)_ = 9.268, *p* = 0.008; treatment: *F*_(1, 15)_ = 3.347, *p* = 0.087; interaction: *F*_(1, 15)_ = 3.023, *p* = 0.013]. When controlling for remainder of the telencephalon volume, we found that dorsal cortical volume was not significantly affected by the covariate [*F*_(1, 13)_ = 0.573, *p* = 0.462], territorial status [*F*_(1, 13)_ = 0.015, *p* = 0.904], treatment [*F*_(1, 13)_ = 0.022, *p* = 0.886], or the interaction [*F*_(1, 13)_ = 134, *p* = 0.720]; analyses without the covariate yielded similar results [territorial status: *F*_(1, 16)_ = 0.484, *p* = 0.497; treatment: *F*_(1, 16)_ = 0.002, *p* = 0.963; interaction: *F*_(1, 16)_ = 0.627, *p* = 0.440]. Medial cortical volume, when adjusted for volume of the remainder of the telencephalon, significantly differed based on the covariate [*F*_(1, 13)_ = 7.418, *p* = 0.017] but was not significantly affected by the other variables [territorial status: *F*_(1, 13)_ = 0.285, *p* = 0.602; treatment: *F*_(1, 13)_ = 0.141, *p* = 0.741; interaction: *F*_(1, 13)_ = 4.235, *p* = 0.06]. However, when volume of remainder of the telencephalon was not included as a covariate, there was a significant effect of territorial status [*F*_(1, 16)_ = 5.505, *p* = 0.032] and the interaction between territorial status and treatment [*F*_(1, 14)_ = 7.635, *p* = 0.014] on medial cortical volume; treatment alone was not significant [*F*_(1, 14)_ = 0.587, *p* = 0.455] (Figure 6).

**Figure 7:**
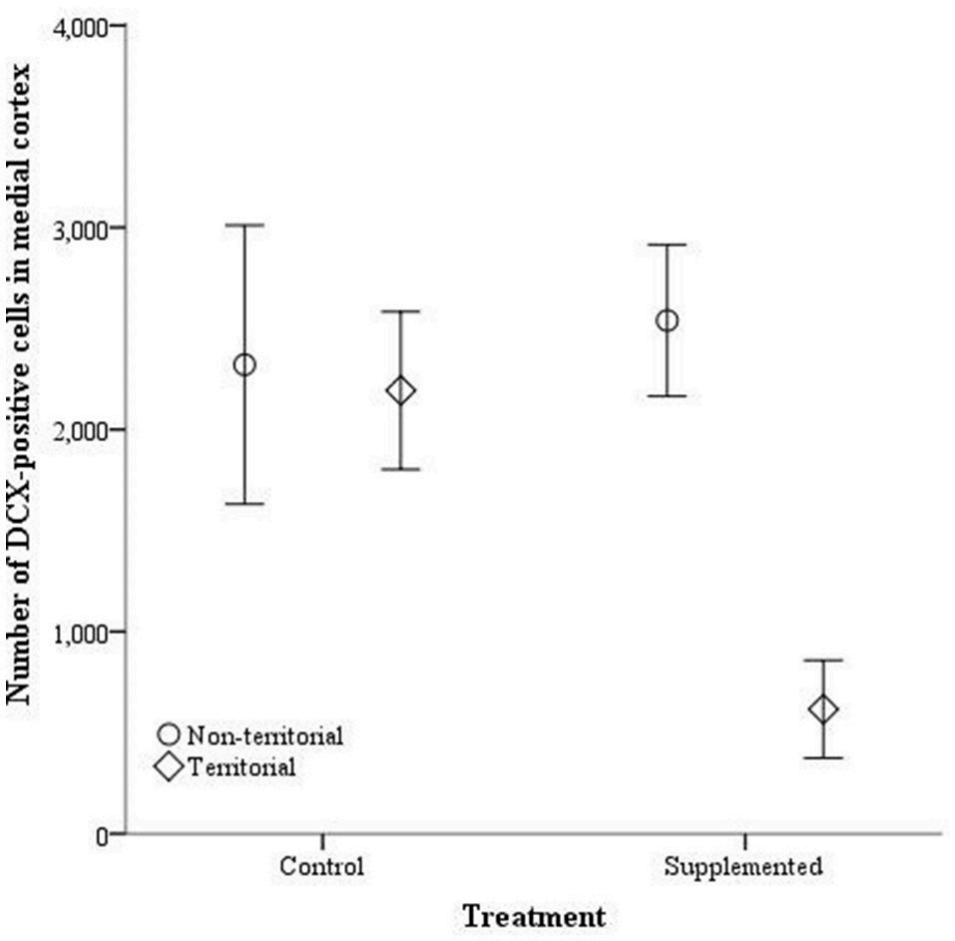
**The total number of doublecortin-positive cells in the medial cortex ± SEM in non-territorial (open circles) and territorial (diamonds) male side-blotched lizards (*Uta stansburiana*)**. Individuals were either subjected to testosterone implants (supplemented) or an empty vehicle (control) for 2 months. Supplemented territorial males had fewer doublecortin-positive cells in the medial cortex compared with the other groups (*p* = 0.05).

The number of doublecortin-positive cells did not significantly differ in the dorsal cortex for any of the independent variables, either with dorsal cortical volume included as a covariate [dorsal cortex volume: *F*_(1, 14)_ = 3.764, *p* = 0.074; territorial status: *F*_(1, 14)_ = 0.027, *p* = 0.872; treatment: *F*_(1, 14)_ = 1.203, *p* = 0.293; interaction: *F*_(1, 14)_ = 4.27, *p* = 0.059] or without [territorial status: *F*_(1, 15)_ = 0.063, *p* = 0.805; treatment: *F*_(1, 15)_ = 629, *p* = 0.440; interaction: *F*_(1, 13)_ = 3.726, *p* = 0.073]. Similarly, the number of doublecortin-positive cells in the medial cortex, when medial cortical volume was included as a covariate, was not significantly affected by the covariate [*F*_(1,14)_ = 0.067, *p* = 0.799], territorial status [*F*_(1, 14)_ = 3.780, *p* = 0.072], treatment [*F*_(1, 14)_ = 2.453, *p* = 0.140], or the interaction between territorial status and treatment [*F*_(1, 14)_ = 2.756, *p* = 0.119]. However, when the covariate was not included, territorial status [*F*_(1, 16)_ = 5.891, *p* = 0.027] and the interaction between territorial status and treatment [*F*_(1, 16)_ = 4.513, *p* = 0.05] significantly affected the number of doublecortin-positive cells in the medial cortex while treatment alone was not a significant predictor [*F*_(1, 16)_ = 2.586, *p* = 0.127] (Figure 7).

## Discussion

### The effects of testosterone on the brain

We found that testosterone supplementation had a direct effect on brain attributes outside of differential environmental experiences. Two months after testosterone implantation, telencephalon volume, medial cortical volume, and the total number of doublecortin-positive cells in the medial cortex were downregulated in adult territorial males despite no concurrent change in spatial environmental experiences. Interestingly, controlling for the telencephalon volume in analyses of medial cortical volume and controlling for the medial cortical volume in the analyses of the total number of new neurons (which effectively analyses neuron density) rendered all analyses non-significant, suggesting that variation in absolute volume and the production of new neurons within the medial cortex is likely a product of proportional downregulation of neural attributes more globally across the entire telencephalon. There were no significant effects of elevated testosterone on any measurements of the dorsal cortex, regardless of covariate inclusion or exclusion in the analyses.

Although we determined that testosterone can directly modulate medial cortical attributes, it was not in the direction predicted. Many studies, primarily in rodent and bird models, have demonstrated that increased testosterone levels upregulate volume and neurogenesis in certain brain regions. For instance, seasonal changes in testosterone have been positively correlated with changes in brain attributes (e.g., Rasika et al., 1994; Galea et al., 2006) and reproductively active males demonstrate increased new neuron survival compared with reproductively inactive males (e.g., Ormerod and Galea, 2003), both of which suggest that testosterone may be one potential mechanism directly and positively regulating brain attributes. Experimental evidence tends to support the positive correlation between testosterone and brain plasticity; testosterone supplementation has been associated with increased volume and survival of new neurons in particular brain regions (Rasika et al., 1994; Alvarez-Buylla and Kirn, 1997; Galea et al., 1999, 2006, 2008, 2013; Absil et al., 2003; Spritzer and Galea, 2007; Galea, 2008; Balthazart and Ball, 2016). However, some studies have found that testosterone supplementation has no effect on neurogenesis (Pfau et al., 2010; Zhang et al., 2010; Carrier and Kabbaj, 2012) and, in some cases, testosterone suppresses neurogenesis (Brannvall et al., 2005; Estrada et al., 2006; Buwalda et al., 2010; Allen et al., 2014).

Although these results are seemingly contradictory, methodological factors may contribute to the discordance. For instance, Hamson et al. (2013) suggested that, in rodent models, testosterone's positive effects on hippocampal neurogenesis may occur between 16 and 30 days, whereas elevated testosterone exposure outside of that window may have detrimental effects, indicating that testosterone supplementation may have differential effects based on the duration of exposure (Buwalda et al., 2010; Spritzer et al., 2011). Further, the effects of testosterone supplementation can be dependent on dosage (e.g., Galea et al., 1999), route of administration (e.g., injection, implants), timing of supplementation vs. application of an exogenous neurogenesis marker (Spritzer and Galea, 2007; Allen et al., 2014), interact with environmental cues/seasonality (e.g., Moore, 1988; Bernard and Ball, 1997; O'Bryant and Wilczynski, 2010), and may differ based on species (e.g., Powers, 2016). Previous studies have used testosterone supplementation that was shorter in duration (<2 months) and no previous study has used a reptilian species to examine the effects of testosterone on neurogenesis. Further, our study utilized doublecortin, an endogenous marker that generates a combined measure of proliferation and survival (Rao and Shetty, 2004), precluding separation of the two. Our study confirmed that testosterone supplementation caused a decrease in doublecortin expression in territorial males but testosterone may have downregulated neuronal proliferation while not affecting, or even positively affecting, neuron survival. Utilization of an exogenous label, such as BrdU, and sacrificing at various time points would allow for detangling the contribution of cellular proliferation vs. survival rates to overall neurogenesis rates. Alternatively, utilization of an endogenous marker expressed only during proliferation would elucidate the effects of testosterone exclusively on the proliferative stage of neurogenesis. Because our and previous studies differ on many of the aforementioned methodological components, generalizability of the effects of testosterone on the brain remains equivocal.

Interestingly, while testosterone supplementation within the laboratory setting was sufficient to elevate testosterone levels and induce neural plasticity, testosterone levels are at least four times higher in the field than what we found in the current study (average 48–203 ng/mL in the field [variation based on morphotype and season, Sinervo et al., 2000] vs. average 11.3 ng/mL in supplemented individuals in the current study). Further, previous literature suggests that our implants should have elevated and maintained testosterone at physiologically-relevant levels in this species (DeNardo and Licht, 1993; DeNardo and Sinervo, 1994). However, our study differed in that our supplementation treatment lasted 2 months; no previous studies in this species have examined supplemented hormone levels past 1 month thus the effects of longer-term supplementation are unknown. Additionally, in ectothermic taxonomic groups, individuals subjected to higher temperatures are thought to clear testosterone quicker (e.g., Pearson et al., 1976; Krohmer et al., 1987). We maintained ideal thermoregulatory conditions within the laboratory; temperatures were consistent from day to day and basking temperatures were higher than the average temperatures at the field site for April and May. Thus, it is possible that the higher average temperatures found in the laboratory were sufficient to clear the supplemented testosterone faster in our animals. Alternatively, but unlikely, all of our implants could have released large amount of testosterones very early while releasing less toward the end of the treatment. While we could have taken blood samples more frequently to assess testosterone levels throughout the 2 month experimental time frame, oversampling would have induced stress in our animals which is a strong down regulator of neurogenesis (e.g., Schoenfeld and Gould, 2012). Regardless of the consistency of testosterone release, the testosterone levels were significantly higher in implanted animals at the end of our 2 month treatment indicating that the implants were functional. Altering methodological protocol to artificially elevate testosterone levels to that seen in the field should be essential for future studies; doing so may yield different results that found in the current study.

Considering that we did not castrate our subjects, endogenous levels of testosterone should have also contributed to overall circulating levels of testosterone. However, environmental components, particularly in the laboratory, can affect homeostatic control of testosterone levels. For instance, previous studies have found that endogenous testosterone levels are typically lower in laboratory-reared species, including reptiles, and this may be due to environmental factors in the laboratory that differ from those in the field (e.g., Callard et al., 1976; Licht et al., 1985; Krohmer et al., 1987; Moore et al., 1991; Soto-Gamboa et al., 2005; Calisi and Bentley, 2009). Changes in day length have often been shown to be important in inducing changes in testosterone levels, reproductive physiology, and behavior, particularly in the laboratory (e.g., Wingfield and Kenagy, 1991). However, our changes in day length mimicked ambient and thus was likely not the modulator of these differences. Further, many reptiles do not demonstrate reproductive responses to changes in photoperiod (e.g., Ferrell, 1984; Moore et al., 1984). Temperature appears to be more important to changes in reproductive physiology (e.g., Pearson et al., 1976; Krohmer et al., 1987) and we maintained temperatures that should have been ideal for upregulating testosterone production. An alternative possibility is that our subjects were not allowed social interactions during the protocol yet social interactions are important modulators of androgen levels in this species (Wingfield et al., 1990). Males in our study were raised in isolation while males in the field typically engage in agonistic interactions with other males and come in contact with many females during the breeding season (e.g., Zamudio and Sinervo, 2000). Because of the lack of opportunity for territorial and reproductive behaviors in our study, we would expect testosterone levels to be much lower than those found in the field. While it is unclear what caused either a downregulation in testosterone after the 2 months treatment or did not allow supplementation to achieve levels seen in the field, even a modest elevation of testosterone within the laboratory setting was still sufficient to downregulate telencephalon volume, medial cortex volume, and neurogenesis in supplemented territorial males. It is not, however, unreasonable to expect that testosterone levels that more closely parallel levels in the field would have more dramatic effects on neural attributes.

While testosterone downregulated cortical attributes in the medial cortex of territorial males, it had no effect on any of the measured neural attributes in the dorsal cortex, whether the covariate was included or not, suggesting that the effects of testosterone may be region-specific. In support of this, previous studies have found that testosterone differentially modulates volume in different brain regions in birds and lizards (Balthazart et al., 2008; Small et al., 2015; Wilson, 2015; Balthazart and Ball, 2016) and only increases cell proliferation in the ventricular zone in canaries (Barker et al., 2014) and the amygdala in rodents (Fowler et al., 2003). While both the medial and dorsal cortices are involved with spatial cognition, they serve slightly different functions—the medial cortex concerns spatial learning and the dorsal cortex is involved in spatial mapping (Grisham and Powers, 1990; Petrillo et al., 1994; Reiman Avigan and Schade Powers, 1995; Rodríguez et al., 2002; López et al., 2003). This suggests that the effects of testosterone could differ based on structural and functional differences between the medial and dorsal cortices. Further, the medial and dorsal cortices vary in the absolute number of new neurons as well as modulation of new neuron production rates. More new neurons are produced and incorporated into the medial cortex (Font et al., 2002) and spatial use experiences only upregulate new neuron production in the medial cortex (LaDage et al., 2013). Thus, while steroid hormones can cross the blood-brain barrier, certain regions of the brain, particularly in regions demonstrating more plasticity, may be more susceptible to testosterone and other hormonal influences.

The effects of testosterone on the brain has been shown to occur through an androgen-specific mechanism (e.g., Spritzer and Galea, 2007), possibly through androgen receptors (ARs) (e.g., Galea, 2008). Nuclear ARs are commonly found in areas associated with reproductive behavior (e.g., Simerly et al., 1990). Some other areas of the brain, including the cortices in lizards, are also AR-immunoreactive suggesting that testosterone could possibly have a direct effect on brain attributes in these regions as well (e.g., Simerly et al., 1990; Clancy et al., 1992; Young et al., 1994; Moga et al., 2000; Rosen et al., 2002; Sarkey et al., 2008). In a recent study, Hamson et al. (2013) demonstrated that the survival of new neurons in the mammalian dentate gyrus can be regulated by testosterone and that this occurs via ARs. However, new neurons in the dentate gyrus did not express ARs suggesting that testosterone indirectly modulates neurogenesis in this area, possible via other hippocampal regions that contain high densities of ARs and are known to have regulatory effects on neurogenesis (Hamson et al., 2013). Interestingly, Brannvall et al. (2005) also demonstrated that androgens act through ARs to modulate neurogenesis but, unlike the previous study, they found that neural stem cells within the dentate gyrus did express ARs, suggesting a possible direct effect of testosterone on proliferating cells in that area. Unfortunately, their methodology precluded identification of these cells as neurons or glia. Thus, while testosterone is correlated with neurogenesis, it is still equivocal if the effect of testosterone is direct and mediated through ARs on new neurons. Testosterone could also have indirect effects as it can be converted to estradiol and several other reduced androgens. Thus, the effect of testosterone could be quite broad and mediated through other signaling mechanisms that could affect neurogenesis and other neural attributes (e.g., Rasika et al., 1999; Harley et al., 2000; Smith et al., 2002; Hebbard et al., 2003; MacLusky et al., 2006; Hamson et al., 2013; Allen et al., 2014).

### Territorial predisposition modulates the relationship between testosterone and the brain

In some species, males that adopt alternative mating strategies also have variation in testosterone levels that can differently organize or activate the nervous system (e.g., Marler et al., 1999; Rhen and Crews, 2002; Aubin-Horth et al., 2005; Crews and Moore, 2005; Bass and Forlano, 2008; Schunter et al., 2014). While we found that testosterone implants elevated testosterone levels similarly between territorial and non-territorial males, testosterone-implanted non-territorial males demonstrated no detectable changes in cortical attributes when compared with control groups while supplemented territorial males downregulated cortical attributes. Thus, testosterone supplementation appears to differentially affect males contingent upon territorial predisposition, despite territorial and non-territorial males having similar beginning and end testosterone levels. In the field, territorial predisposition, testosterone, and spatial area use co-vary; territorial males have naturally elevated testosterone levels compared with non-territorial males (Sinervo et al., 2000) and testosterone is associated with increased activity and home range size (e.g., DeNardo and Sinervo, 1994; Sinervo et al., 2000). Also, previous studies in this species have determined that territorial predisposition modulates the differential effects of testosterone on other physiological measures. For instance, increasing testosterone increases endurance and sprint speed in non-territorial but not territorial males suggesting that territorial males are at their physiological limits for these variables (Mills et al., 2008). Also, our previous work demonstrated that increasing spatial area use increased rates of neurogenesis in territorial males but not in non-territorial males (LaDage et al., 2013). Thus, territorial predisposition correlates with differences in a variety of physiological measures including neural plasticity.

Other physiological factors, such as differences in homeostatic mechanisms, target tissue response, or synergy with other hormones may also contribute to the differential morphotypic modulation of neural attributes, yet it is still unclear how these factors interact with testosterone and morphotype to produce the neural phenotype. For example, Knapp (2003) proposed that non-territorial morphs may be insensitive to testosterone relative to territorial males due to fewer androgen receptors, which would support our results that non-territorial males did not differ from controls despite having elevated testosterone levels. This explanation, however, does not explain reduced neural attributes in territorial males. An alternative explanation, but not mutually exclusive, concerns testosterone and its possible morphotypic-specific interactions with other hormones. Testosterone and social behaviors demonstrate a reciprocal positive relationship with each other but an increase in both also correlates with increased glucocorticoid release, presumably to mobilize energy stores for reproductive behaviors (e.g., Emerson and Hess, 2001; Moore and Jessop, 2003; Wilczynski et al., 2005). However, chronically high levels of glucocorticoids can subsequently suppress testosterone and social behaviors, likely for energy recovery (e.g., Emerson and Hess, 2001). Previous studies have also demonstrated that the relationship between testosterone and glucocorticoids can also be modulated by territorial status. In tree lizards (*Urosaurus ornatus*), both territorial and non-territorial males increase glucocorticoids in response to a stressor. However, only non-territorial males demonstrated a concurrent decrease in testosterone, indicating different downstream homeostatic responses to elevated glucocorticoids (Knapp and Moore, 1995, 1996, 1997). In rodent models, high levels of glucocorticoids strongly downregulate hippocampal neurogenesis (reviewed in Schoenfeld and Gould, 2012; Saaltink and Vreugdenhil, 2014), thus there may be a link among increased testosterone, increased glucocorticoids, and decreased neurogenesis and this may be modulated by territorial predisposition. Territorial and non-territorial males may differ in sensitivity to elevated levels of glucocorticoids, possibly via differential expression of glucocorticoid receptors or differences in homeostatic stress reactivity (e.g., LaDage, 2015), which would in turn differentially modulate rates of neurogenesis. In the current study, if increased testosterone concurrently increased glucocorticoids and if territorial males were unable to downregulate glucocorticoids in the face of constantly elevated testosterone while non-territorial males could, it would not be surprising that territorial males demonstrated downregulated neural attributes while non-territorial males did not. However, we did not measure circulating glucocorticoid levels or glucocorticoid receptor expression therefore the relationship between testosterone, glucocorticoids, and the brain remains to be tested.

## Conclusions

We found that supplementing testosterone, outside of variation in spatial use experiences, was sufficient to directly downregulate telencephalon volume, medial cortical volume and neurogenesis. Analyses controlling for the overall telencephalon or medial cortex volumes indicated that medial cortical volume and neurogenesis may correlate with more global downregulated changes in the brain as the densities of new neurons were not different between treatment and control while the total numbers were. Overall, these results are counter to previous studies in rodents demonstrating that testosterone upregulates neural attributes but this discrepancy could be a result of methodology differences. As of now, there is a paucity of literature on the effects of testosterone on the hippocampus, particularly in non-model species (e.g., Powers, 2016), thus precluding meaningful generalizations at this point. Interestingly, downregulation of cortical attributes was only seen in territorial males indicating that territorial predisposition can modulate the effects of testosterone on the brain. Although morphotypes may demonstrate similar levels of testosterone, they may differ in response to such (e.g., Moore, 1988; Baird and Hews, 2007) leading to morphotypic variation in neuroendocrine pathways, downstream physiological effects, and subsequent behaviors (e.g., Knapp, 2003). Presumably, morphotypic-specific responses to testosterone are based on naturally selected behaviors and life histories that confer differential fitness benefits for morphotypes (e.g., Crews and Moore, 2005).

Studies that examine the mechanistic basis of the effects of testosterone on the brain could elucidate if testosterone, estradiol, and reduced androgens act directly, through ARs on new neurons, or indirectly to control the production and survival of new neurons as well as other forms of brain plasticity. From a functional perspective, it is still unknown if testosterone is sufficient to induce differences in spatial memory ability between territorial and non-territorial males and, if so, whether neurogenesis is a necessary process used to encode those spatial memories. Similarly, differential spatial use or social experiences may also induce changes in brain plasticity differently than that found in studies that constrain those experiences. Ultimately, the current study underscores the importance of understanding how naturally-selected morphotypic differences in the neuroendocrine axis may alter neural plasticity in ways that could not be predicted from clinical studies in rodent models. Understanding the interplay among circulating hormones, spatial use, and polymorphic differences would aid our understanding of the control and functional significance of neurogenesis and other forms of brain plasticity.

## Author contributions

LL, VP, and BS designed the experiments. LL, TR, and CD performed the experiments. LL performed the immunohistochemistry and stereology. LL, TR, and VP analyzed and interpreted the data. LL drafted the paper and all authors critically reviewed, revised, and approved the final version of the paper.

## Funding

This research was supported by a National Science Foundation award to LL, VP, and BS (IOS-0918268). VP was also supported by the National Science Foundation award (IOS-1351295).

### Conflict of interest statement

The authors declare that the research was conducted in the absence of any commercial or financial relationships that could be construed as a potential conflict of interest. The reviewer PP and handling Editor declared their shared affiliation, and the handling Editor states that the process nevertheless met the standards of a fair and objective review.
